# Myocardial Injury Is Associated with the Incidence of Major Adverse Cardiac Events in Patients with Severe Trauma

**DOI:** 10.3390/jcm11247432

**Published:** 2022-12-15

**Authors:** Alexandra Stroda, Carina Jaekel, René M’Pembele, Alexander Guenther, Theresa Tenge, Carl Maximilian Thielmann, Simon Thelen, Erik Schiffner, Dan Bieler, Michael Bernhard, Ragnar Huhn, Giovanna Lurati Buse, Sebastian Roth

**Affiliations:** 1 Department of Anesthesiology, Medical Faculty, University Hospital Duesseldorf, Heinrich-Heine-University Duesseldorf, 40225 Dusseldorf, Germany; 2 Department of Orthopedics and Trauma Surgery, Medical Faculty, University Hospital Duesseldorf, Heinrich-Heine-University Duesseldorf, 40225 Dusseldorf, Germany; 3 Department of Dermatology, University Hospital Essen, University of Duisburg-Essen, 45147 Essen, Germany; 4 German Cancer Consortium (DKTK), 69120 Heidelberg, Germany; 5 Emergency Department, Medical Faculty, University Hospital Duesseldorf, Heinrich-Heine-University Duesseldorf, 40225 Dusseldorf, Germany; 6 Department of Anesthesiology, Kerckhoff Heart and Lung Center, 61231 Bad Nauheim, Germany

**Keywords:** myocardial ischemia, cardiovascular complications, hemodynamics, prognosis, multiple trauma

## Abstract

Background: Severe trauma potentially results in end-organ damage such as myocardial injury. Data suggest that myocardial injury is associated with increased mortality in this cohort, but the association with the incidence of in-hospital major adverse cardiac events (MACE) remains undetermined. Methods: Retrospective cohort study including adult patients with severe trauma treated at the University Hospital Duesseldorf between January 2016 and December 2019. The main exposure was myocardial injury at presentation. Endpoints were in-hospital incidence of MACE and incidence of acute kidney injury (AKI) within 72 h. Discrimination of hsTnT for MACE and AKI was examined by the receiver operating characteristic curve (ROC) and the area under the curve (AUC). We conducted multivariate logistic regression analysis. Results: We included 353 patients in our final analysis (72.5% male (256/353), age: 55 ± 21 years). The AUC for hsTnT and MACE was 0.68 [95% confidence interval (CI): 0.59–0.78]. The AUC for hsTnT and AKI was 0.64 [95% (CI): 0.55–0.72]. The adjusted odds ratio (OR) for myocardial injury and MACE was 2.97 [95% (CI): 1.31–6.72], and it was 2.14 [95% (CI): 1.03–4.46] for myocardial injury and AKI. Conclusion: Myocardial injury at presentation in patients with severe trauma is independently associated with the incidence of in-hospital MACE and AKI.

## 1. Introduction

Severe trauma is among the leading causes of death in the population of both males and females below 40 years old [1]. The overall patient outcome is not solely dependent on survival of the initial trauma; it rather consists of a variety of other factors impacting prognosis. Besides obvious physical injuries, various pathophysiological mechanisms can cause secondary alterations during severe trauma [2]. For example, hemorrhage may ultimately create a mismatch between oxygen supply and demand, potentially resulting in a vicious cycle with end-organ damage [3,4,5]. This implies that, at a cellular level, insufficient oxygen delivery results in anaerobic metabolism with the accumulation of lactate acid, inorganic phosphates, and oxygen radicals, which—at the end stage—may lead to apoptosis [6]. In addition, bleeding causes vasoconstriction or, in the worst case, exsanguination with pulselessness, which increases tissue hypoxia [5]. In our previous work, we showed that myocardial injury in the setting of severe trauma is a common complication, directly associated with increased mortality, regardless of its cause [7]. Current evidence from a perioperative setting, even in noncardiac surgery, suggests that perioperative myocardial injury is associated with complications such as major cardiac adverse events (MACE), occurring in roughly 15% of patients undergoing noncardiac surgery [8].

A recent analysis within the group of patients with severe trauma linked myocardial injury to in-hospital mortality. However, the association between myocardial injury at initial presentation and the incidence of in-hospital MACE is not yet investigated [7]. Thus, our aim was to investigate a potential association among myocardial injury and MACE in patients with severe trauma.

## 2. Materials and Methods

We conducted a retrospective single-center cohort study. Before study initiation, approval was granted by the local institutional review board (reference number 2020-1122). All handling of personal data fulfilled the conditions of the General Data Protection Regulation (EU) 2016/679 and complied with the GCP Guidelines. Due to the retrospective nature of this study, the need for written informed consent could be waived. We generated the research question based on the PICO format. This study was performed in accordance to the declaration of Helsinki.

### 2.1. Study Population

Inclusion criteria were as follows: adult patients ≥ 18 years, suffering from severe trauma, defined as Injury Severity Score (ISS) ≥ 16, admitted to the emergency department of the University Hospital Duesseldorf between January 2016 and December 2019. Patients were only included when the trauma occurred immediately before the emergency service was called. In addition, we did not include patients that were transferred from another hospital to avoid the results being influenced by different troponin dynamics. We also excluded patients with missing troponin values at presentation. 

### 2.2. Outcome Measures 

The primary endpoint was the in-hospital incidence of major adverse cardiac events (MACE). This composite endpoint included nonfatal cardiac arrest, acute myocardial infarction, new onset of cardiac arrhythmia, and stroke [8]. Acute myocardial infarction was defined according to the fourth universal definition [9]. Cardiac arrhythmias were defined as any new and hemodynamically relevant arrhythmia with the need for pharmacological or electrical treatment [8]. Stroke was defined according to the guidelines of the American Heart Association (AHA). In-hospital MACE was assumed, when listed in the patients’ medical record. The plausibility of the diagnosis was then validated by a trained member of the study team.

The secondary endpoint was the occurrence of acute kidney injury (AKI) within 72 h after hospital admission. AKI was defined according to the “Kidney Disease Improving Global Outcomes” Working Group (KDIGO) as an increase in serum creatinine either at least 1.5 times from baseline or a 0.3 mg/dL increase from baseline [10].

### 2.3. Statistical Analysis

Statistical analysis was performed using SPSS 27.0. We conducted a complete case analysis. Continuous data are shown as the median (interquartile range (IQR)), whereas categorical data are shown as the mean ± standard deviation (SD). The discrimination of highly sensitive troponin T (hsTnT) for in-hospital MACE was examined by the receiver operating characteristic curve (ROC) and the area under the curve (AUC). The same was performed to investigate the discrimination of hsTnT for AKI. Afterwards, we created two multivariate logistic regression models with forced entry of predefined covariables to examine the independent association of hsTnT and MACE and AKI, respectively.

### 2.4. Independent Variable and Covariates

The independent variable of the above-mentioned ROC curves was myocardial injury, defined according to the 4th universal definition of myocardial injury as elevated hsTnT (Roche Diagnostics, Elecsys^®^, Rotkreuz, Switzerland) above the 99th percentile (hsTnT > 14 ng/mL) [9].

Regarding the multivariate logistic regression models, we could choose one covariate per 10 events, as prescribed by the rule of thumb [11]. Based on the current literature, we included the following covariables for the association of myocardial injury and MACE: age and coronary artery disease (CAD). CAD was assumed when diagnosed via coronary angiography, including any type and severity. For the association of myocardial injury and AKI, we chose age, sex, and severe chronic kidney disease (CKD), defined as CKD ≥ III according to the KDIGO criteria, respectively.

To examine the association of either MACE, AKI, or the combination of both with mortality, we divided the cohort into four risk groups (patients without MACE and AKI, patients with only MACE, patients with only AKI, and patients with both MACE and AKI) and calculated mortality rates for each group. Afterwards, we performed multivariate logistic regression analysis to calculate odds ratios.

## 3. Results

**Figure 1:**
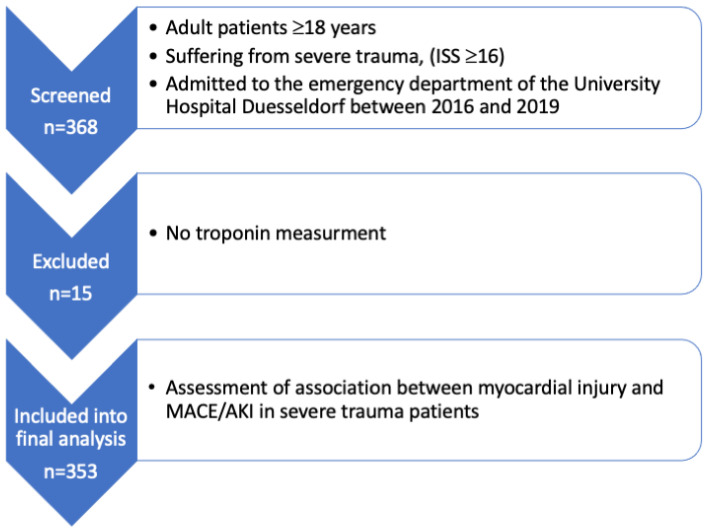
Study flow chart showing the selection process of the study. ISS *=* Injury Severity Score; MACE = major adverse cardiac events; AKI = acute kidney injury.

Out of 368 patients screened, 353 patients were included in our final analysis (72.5% male (256/353), age: 55 ± 21 years). Figure 1 shows the study flow chart. In total, 149 patients exhibited myocardial injury at initial presentation, 32 patients (9.0%) developed an in-hospital MACE, and, in 42 patients (11.9%), AKI occurred within 72 h. All-cause in-hospital mortality was 26% (92/353). Moreover, 22 patients with myocardial injury at presentation developed in-hospital MACE (14.8%), whereas 10 patients without myocardial injury developed MACE (4.9%). Table 1 shows detailed patient characteristics.

### 3.1. Discrimination of Myocardial Injury for MACE and AKI

**Figure 2:**
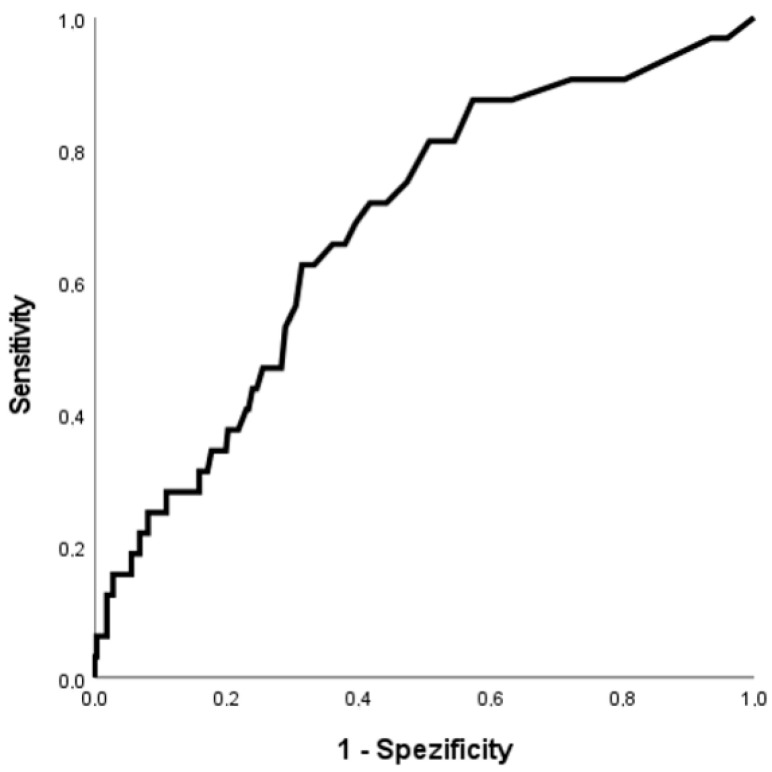
Receiver operating characteristic (ROC) curve showing the discrimination of initial highly sensitive troponin T (hsTnT) for major adverse cardiac events.

**Figure 3:**
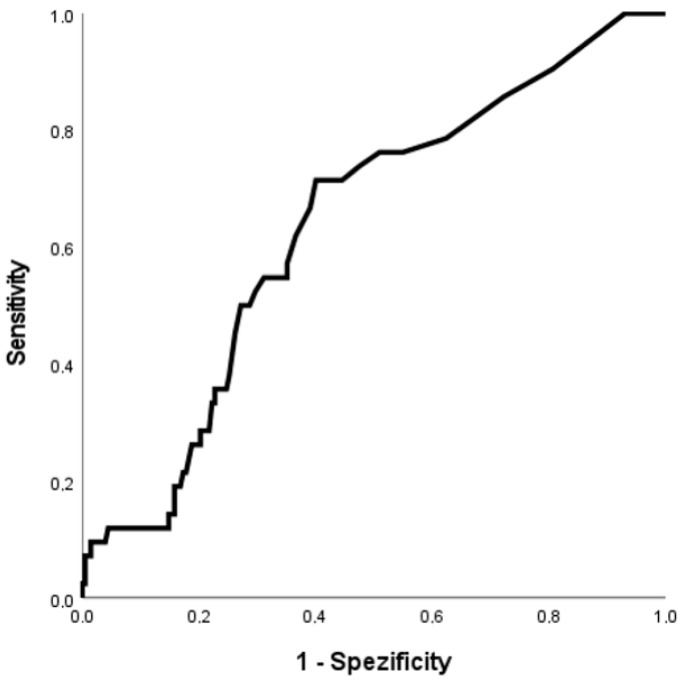
Receiver operating characteristic (ROC) curve showing the discrimination of initial highly sensitive troponin T (hsTnT) for AKI within 72 h.

The ROC curve for the discrimination of hsTnT for MACE is shown in Figure 2. The AUC was 0.68 [95% confidence interval (CI): 0.59–0.78]. The univariate regression model for the association of myocardial injury and MACE revealed an odds ratio (OR) of 3.36 [95% (CI): 1.54–7.33]. Figure 3 shows the ROC curve for the discrimination of hsTnT and AKI. The AUC was 0.64 [95% (CI): 0.55–0.72]. The odds ratio based on the univariate regression model was 2.81 [95% (CI): 1.42–5.58].

### 3.2. Multivariate Regression Models

After the forced entry of predefined covariables, the OR for the association between myocardial injury and MACE remained significant (OR: 2.97 [95% (CI): 1.31–6.72]). Full results of the multivariate logistic regression analysis for myocardial injury and MACE are shown in Table 2. The adjusted OR for the association between myocardial injury and AKI was 2.14 [95% (CI): 1.03–4.46]. Results of this model are shown in Table 3. During the review process, we additionally included the Injury Severity Score into the multivariate model for myocardial injury and MACE (see Appendix A, Table A1).

### 3.3. Association between MACE, AKI, and Mortality in Patients with Severe Trauma

Mortality for patients without MACE and AKI was 6.4% (12/187), whereas mortality in patients with MACE and AKI was 80% (8/10). Results of the further regression analysis are shown in Table 4.

## 4. Discussion

In this study, we showed the independent association of myocardial injury at presentation with the incidence of in-hospital MACE and AKI within 72 h of admission in patients with severe trauma (defined as Injury Severity Score (ISS) ≥ 16 upon admission).

### 4.1. MACE and Severe Trauma

Until now, there has been limited evidence concerning the incidence and causal factors of MACE in patients with severe trauma. As we could identify a 9% incidence of MACE in our cohort, our study was in line with previous studies [12,13,14]. Naganathar et al. analyzed a cohort of 300 trauma patients prospectively and the incidence of adverse cardiac events (ACE), defined as arrhythmias, infarctions, cardiac failure, and angina, and its association with cardiac biomarkers, especially human-heart-type fatty acid binding protein (hFABP), was investigated. Patients were included independently of ISS and the median ISS in this cohort was 19.5. This difference suggests that injuries were more severe in the present cohort. They were able to show an association between hFABP-based cardiac injury and adverse cardiac events, but did not investigate troponin as the most established biomarker of myocardial injury in clinical routine [12].

In two studies by De’Ath et al., 135 trauma patients were investigated retrospectively. First, they investigated the impact of trauma-induced secondary cardiac injury (TICSI) on the patients’ outcomes. Second, they revealed that elevated inflammatory cytokines were associated with the development of TICSI. Cardiac biomarkers at admission (in their case, hFABP and BNP) were higher in patients with ACE, which is in line with our results. In these studies, ACE was defined as cardiac death, myocardial infarction, angina, arrhythmia, and cardiogenic shock. However, multivariate logistic regression analysis was not performed and therefore an independent association between cardiac biomarkers and ACE could not be identified [13,15].

### 4.2. Clinical Relevance

In order to improve the outcomes of patients suffering from severe trauma besides surviving the initial trauma, the identification of risk factors for subsequent complications up to and including death is crucial. With this study, we were able identify myocardial injury at presentation as an independent risk factor for MACE. In a recent study, we showed that hypotension during treatment in the resuscitation room is independently associated with myocardial injury in patients with severe trauma [16]. Thus, maintaining a mean arterial pressure ≥ 65 mmHg could act as a potential lever in the prevention of MACE. Moreover, we could show that mortality in patients suffering from both MACE and AKI is much higher than in patients without these complications or with only one of these complications.

In the non-cardiac-surgery setting, the role of MACE has already been widely investigated. The incidence of MACE in this setting amounts to 10%, which is similar to the results of our study [8]. As the independent association of myocardial injury and MACE in patients undergoing non-cardiac surgery has been proven, several possibilities of prevention have been suggested and/or already investigated in the past. There is evidence that pharmacological therapy using aspirin or statins could serve as secondary prevention of MACE in this setting [17,18]. Furthermore, observational studies suggested that several perioperative factors, when optimized, could prevent postoperative myocardial injury and MACE. These include, for example, perioperative basic quality of care and the prevention of hypothermia, hypoxemia, anemia, pain, tachycardia, hypotension, and hypoglycemia [19,20,21]. In future studies, the transferability of these protective factors to the severe trauma setting should be investigated.

### 4.3. Strengths and Limitations

There are several limitations to the present study. Among this study’s limitations is its retrospective nature. However, data for the initial database were collected prospectively. Consequently, some patient data were not available, and we had to exclude patients without troponin data at arrival. However, this only applied to a small number of patients. Nevertheless, we investigated a large representative cohort of patients with severe trauma. To the best of our knowledge, we are the first to show that the coincidence of MACE and AKI in patients with severe trauma leads to a strong increase in risk for in-hospital mortality. Due to missing data regarding creatinine values within the first 72 h, these findings should be validated in a large prospective study. Moreover, we retrospectively screened the patients’ records for the in-hospital occurrence of MACE, so we could not obtain full details about MACE documentation. Another limitation is that we only investigated troponin as a marker of myocardial injury. Although this is the established standard marker in this context, further studies may also focus on other cardiac biomarkers, such as natriuretic peptides. Finally, we were not able to include all relevant covariables in our multivariate models as the number of events was limited in this study. There may be risk factors (e.g., other cardiovascular comorbidities such as peripheral artery disease, renal insufficiency, or the severity of trauma) that may also relevantly contribute to myocardial injury and MACE.

## 5. Conclusions

The present study shows that myocardial injury is independently associated with the incidence of in-hospital MACE and AKI in patients with severe trauma. These data underline the prognostic relevance of myocardial injury in this specific setting. Future studies should investigate whether the initiation of a secondary prevention measure may help to reduce the incidence of adverse events and thus may improve the outcomes of trauma patients.

## Figures and Tables

**Table 1:** Patient characteristics.

	Patients with Severe Trauma (n = 353)	Patients with Myocardial Injury (n = 149)	Patients without Myocardial Injury (n = 204)	Patients with MACE (n = 32)	Patients without MACE (n = 321)
Baseline characteristics					
Male sex no. (%)	256 (72.5%)	104 (69,8%)	152 (74.5%)	28 (87.5%)	228 (71%)
Age (years)	55 ± 21	62 ± 22	50 ± 18	62 ± 20	54 ± 21
Adipositas (body mass index ≥ 30 kg/m²)	3 (0.8%)	2 (1.3%)	1 (0.5%)	1 (3.1%)	2 (0.6%)
Comorbidities					
Coronary artery disease	28 (7.9%)	16 (10.7%)	12 (5.9%)	5 (15.6%)	23 (7.2%)
Chronic kidney disease (≥CKD III)	9 (2.5%)	7 (4.7%)	2 (1.0%)	2 (6.3%)	7 (2.2%)
Diabetes mellitus	22 (6.2%)	9 (6.0%)	13 (6.5%)	3 (9.4%)	19 (5.9%)
History of arterial hypertension	85 (24.1%)	41 (27.5%)	44 (21.6%)	11 (34.4%)	74 (23.1%)
Peripheral artery disease	5 (1.4%)	3 (2.0%)	2 (1.0%)	1 (3.1%)	4 (1.2%)
ASA physical status					
ASA I	159 (45%)	47 (35.3%)	112 57.1%)	9 (30%)	150 (50.2%)
ASA II	111 (31.4%)	57 (42.9%)	54 27.6%)	13 (43.3%)	98 (32,8%)
ASA III	52 (14.7%)	24 (18%)	28 (14.3%)	8 (26.7%)	44 (14.7%)
ASA IV	7 (2.0%)	5 (3.8%)	2 (1.0%)	/	7 (2.3%)
Trauma-related data					
ISS	28 ± 12 25 (19–33)	30 ± 12 26 (22–38)	26 ± 10 22 (17–29)	32 ± 13 28 (22–40)	27 ± 11 25 (18–33)
GCS at ED arrival	8 ± 5 3 (3–14)	6 ± 5 3 (3–9)	9 ± 5 10 (3–15)	5 ± 4 3 (3–7)	8 ± 5 4 (3–15)
Laboratory values					
Hb (g/dL)	12.3 ± 2.4	11.5 ± 2.5	12.8 ± 2.2	11.9 ± 3.3	12.3 ± 2.3
INR	1.4 ± 0.8	1.6 ± 1.0	1.2 ± 0.5	1.9 ± 1.5	1.3 ± 0.7
PTT (s)	31.7 ± 24.4	38.0 ± 32.6	27.3 ± 15.0	44.1 ± 37.4	30.5 ± 22.4
Base excess	−3.8 ± 5.7	−5.7 ± 6.7	−2.5 ± 4.5	−8.0 ± 7.6	−3.4 ± 5.4
HsTnT initial (ng/mL)	63.3 ± 415.7 11.0 (6.0–28.5)	139.8 ± 633.1 36.0 (21.0–75.0)	7.5 ± 3.0 7.0 (5.0–10.0)	321 ± 1336.9 22 (11.3–76.8)	37.6 ± 98.1 11 (6–26)
Creatinine initial (mg/dL)	1.04 ± 0.62	1.16 ± 0.51 1.1 (0.88–1.3)	0.95 ± 0.68 0.9 (0.73–1.07)	1.15 ± 0.6 1.0 (0.89–1.28)	1.03 ± 0.62 0.98 (0.79–1.1)
Outcome					
Death in hospital	92 (26.1%)	67 (45%)	25 (12.3%)	20 (62.5%)	72 (22.4%)
Myocardial injury	149 (42.2%)	149 (100%)	0 (0%)	22 (68.8%)	127 (39.6%)
In-hospital MACE	32 (9.1%)	22 (14.8%)	10 (4.9%)	32 (100%)	/
Non-fatal cardiac arrest	20 (5.7%)	16 (10.7%	4 (2.0%	20 (62.5%)	/
Myocardial infarction	2 (0.6%)	2 (1.3%)	0 (0%)	2 (6.3%)	/
New-onset arrythmia	9 (2.5%)	5 (3.4%)	4 (2.0%)	9 (28.1%)	/
Stroke	5 (1.4%)	3 (2.0%)	2 (1.0%)	5 (15.6%)	/
AKI ^1^	42 (17.2%)	26 (26.0%)	16 (11.1%)	10 (40%)	32 (14.6%)

Values are presented as N (%) or mean (±SD)/median (IQL), where appropriate; ^1^ 109 missing values. ASA = American Society of Anesthesiologists; ISS = Injury Severity Score; ED = Emergency Department; GCS = Glasgow Coma Scale; Hb = Hemoglobin; INR = International Normalized Ratio; PTT = Partial Thromboplastin Time; HsTnT = Highly Sensitive Troponin.

**Table 2:** Multivariate binary logistic regression model for myocardial injury and in-hospital MACE.

Variable	Regression Coefficient	Odds Ratio	95% Confidence Interval		*p*-Value
			Lower	Upper	
Myocardial injury	1.088	2.97	1.31	6.72	0.009
Age per year	0.007	1.007	0.99	1.03	0.446
Coronary artery disease	0.605	1.83	0.61	5.51	0.282

**Table 3:** Multivariate binary logistic regression model for myocardial injury and AKI within 72 h.

Variable	Regression Coefficient	Odds Ratio	95% Confidence interval		*p*-Value
			Lower	Upper	
Myocardial injury	0.763	2.144	1.031	4.459	0.041
Age per year	0.028	1.029	1.009	1.049	0.003
Sex	−0.74	0.477	0.182	1.253	0.133
CKD * ≥ III	0.848	2.335	0.519	10.511	0.269

* chronic kidney disease.

**Table 4:** Association between MACE, AKI and mortality in patients with severe trauma.

Cohort	Mortality	Odds Ratio (95% CI)	Adjusted Odds Ratio * (95% CI)
Patients without MACE and AKI (n = 187)	N = 12 6.4%	0.064 (0.30–0.138)	0.077 (0.04–0.17)
Patients with MACE (no AKI) (n = 15)	N = 6 40%	2.09 (0.72–6.06)	2.26 (0.73–7.07)
Patients with AKI (no MACE) (n = 32) *	N = 13 40.6%	4.0 (1.80–8.90)	3.19 (1.38–7.36)
Patients with MACE and AKI (n = 10)	N = 8 80%	13.23 (2.75–63.6)	9.15 (1.18–46.20)

* multivariate logistic regression analysis with sex and age as covariates.

## Data Availability

The datasets generated during and/or analyzed for the current study are available from the first author, A.S., on reasonable request.

## Appendix A

**Table A1:** Multivariate binary logistic regression model for myocardial injury and MACE, including the Injury Severity Score (ISS).

Variable	Regression Coefficient	Odds Ratio	95% Confidence Interval		*p*-Value
			Lower	Upper	
Myocardial injury	0.954	2.60	1.13	5.95	0.024
Age per year	0.009	1.009	0.99	1.03	0.349
Coronary artery disease	0.678	1.97	0.65	5.99	0.232
Injury Severity Score	0.024	1.024	0.995	1.054	0.100
